# Two-Party Privacy-Preserving Set Intersection with FHE

**DOI:** 10.3390/e22121339

**Published:** 2020-11-25

**Authors:** Yunlu Cai, Chunming Tang, Qiuxia Xu

**Affiliations:** 1 School of Mathematics and Information Science, Guangzhou University, Guangzhou 510006, China; caiyunlu@gzhu.edu.cn; 2 State Key Laboratory of Cryptology, P.O. Box 5159, Beijing 100878, China; 3 School of Mathematics and Systems Science, Guangdong Polytechnic Normal University, Guangzhou 510665, China; xia_mi0622@126.com

**Keywords:** private set intersection, privacy-preserving, fully homomorphic encryption, secure multiparty computation

## Abstract

A two-party private set intersection allows two parties, the client and the server, to compute an intersection over their private sets, without revealing any information beyond the intersecting elements. We present a novel private set intersection protocol based on Shuhong Gao’s fully homomorphic encryption scheme and prove the security of the protocol in the semi-honest model. We also present a variant of the protocol which is a completely novel construction for computing the intersection based on Bloom filter and fully homomorphic encryption, and the protocol’s complexity is independent of the set size of the client. The security of the protocols relies on the learning with errors and ring learning with error problems. Furthermore, in the cloud with malicious adversaries, the computation of the private set intersection can be outsourced to the cloud service provider without revealing any private information.

## 1. Introduction

In 1978, Rivest first presented the idea of fully homomorphic encryption (FHE) [1]. Gentry constructed the first specific FHE scheme in 2009 [2]. Since then, dramatic progress in FHE is made by Gentry and many other researchers around the world. The first generation is based on an approximate GCD problem of integers and ideal lattices [2,3]; the second generation is based on ring learning with errors (RLWE) and learning with errors (LWE) problems, and developed several techniques, including re-linearization, key switch and modulus reduction, for decreasing noise growth [4,5]; the third generation involves the GSW scheme, which is based on approximate eigenvalues and RLWE [6]. Shuhong Gao’s scheme [7] is a compressed fully homomorphic encryption scheme, denoted by SGFHE below, and this scheme has three features: (1) The cipher with private key encryption is expanded six times and with public key encryption is 10+log2(n), where *n* (a power of 2) is the block length of the message; the computation of all ciphertexts is modulo *r*, where r=16n; and the boundary of noise size is n−1. (2) The bootstrapping algorithm needs only a bootstrapping key and the boundaries of the noise size of the output ciphers are still n−1 with no failure at all. (3) the security of Shuhong Gao’s scheme is based on the learning with errors problems and ring learning with errors problems, and for the block length of any message n≥512, it costs at least $2^{160}$ bit operations for breaking the scheme with the current approaches. In addition, with TFHE bootstrapping [8], the LWE cipher produced could be invalid with a probability of about $2^{-33}$ (for n=500). That probability is very small, and for computing many functions it is useful; however, it cannot be applied to functions that require more than $2^{33}$ bit operations (unless increasing *n*). In SGFHE, the error size of the LWE ciphers after bootstrapping are always bound by n−1; this feature is not available in other FHE schemes. The total time cost for the bootstrapping procedure of the SGFHE scheme is about 130 ms, that is, 10 times as much as TFHE.

Secure multi-party computing (SMPC) is mainly about how to compute a function safely without a trusted third party. Secure multi-party computing was first proposed by Yao Qizhi in 1982. After being developed by Goldreich, Micali, Wigderson et al. [9], secure multi-party computing became a very active research field in modern cryptography. The research on MPC [10] is divided into general schemes and specific schemes designed for certain computing scenarios; the general scheme is not as efficient as a specific optimized scheme that is specially designed for a certain application. In practical applications, specific schemes are more widely used [11]. Secret sharing [12], garbled circuit [13,14], oblivious transfer [15], commitment schemes [16] and homomorphic encryption [17] are the key pieces of technology to realize SMPC, and SMPC is of great significance in the study of secret sharing schemes and privacy protection, where it is widely used in correlation analysis, data security queries, trusted data exchanges, etc. [18,19,20,21,22].

Private preserving set intersection (PSI) computing is an important aspect in secure multi-party computing. It not only performs well in scientific computing, but in real life many data can be represented by sets, so it can be used in privacy protection computing to complete corresponding data computing in the sets. The private preserving set intersection computing is the basic operation in many applications, such as machine learning, data mining [23], secure distributed data connection [24] and in privacy protection law enforcement, where it is especially widely used.

### 1.1. Related Work

Several specialized PSI protocols have been proposed in the literature which are more efficient than using general secure computation [33]. The main methods are: based on oblivious polynomial evaluation [25], based on an oblivious pseudo-random function [26], based on a blind signature [27], based on homomorphic encryption [28], based on the Bloom filter [29], etc. Shen Liyan et al. [30] gave a detailed overview of the development prospects of private preserving set intersection computing, the protocol developed by Google scholar. Mihaela Ion et al. [11] applied private preserving set intersection computing to advertising cooperation.

### 1.2. Contributions

We present three private set intersection protocols. First, we propose a novel private set intersection protocol based on Shuhong Gao’s fully homomorphic encryption scheme and prove the security of the protocol in the honest-but-curious model. We then present a variant of promoted protocol. We also present a variant of the protocol which is a completely novel construction for computing the intersection based on the Bloom filter and a fully homomorphic encryption; this protocol’s complexity is independent of the set size of the client. The security of the protocol relies on the learning with errors and ring learning with errors problems. Furthermore, in a cloud with malicious adversaries, the computation of the private set intersection can be outsourced to the cloud service provider without revealing any private information. The ciphertext extension of the protocols is small so that the protocols have strong practicability.

The remainder of the paper is structured as follows: We next review the basic concepts and techniques used in Section 2. In Section 3, we introduce the homomorphic operation used. We describe the basic two-party computing protocol, the improvement protocol and the two-party computing protocol based on the Bloom filter in Section 4. We present our conclusions in Section 5.

## 2. Basic Concepts and Techniques

### 2.1. Notation

Let χ be an error distribution; according to the distribution χ, x←χ is randomly chosen. For an integer n≥1, let $R_{n}=Z\left[x\right]/\left(x^{n}+1\right)$, $R_{n,q}=Z\left[x\right]/\left(x^{n}+1,q\right)$, where $\left(x^{n}+1,q\right)$ represents the ideal of Z[x] generated by $x^{n}+1$ and *q*. For any polynomial $f\left(x\right)=\sum_{i=0}^{d}f_{i}\left(x^{i}\right)\in R\left(x\right)$, we define the *∞*-norm as ${\left||f\left(x\right)|\right|}_{\infty }=\max_{0\leq i\leq d}\left|f_{i}\right|$.

### 2.2. LWE Ciphers and Modulus Reduction

Regev proposed LWE problem [31,32] over $Z_{q}$. Let χ be a probabilistic distribution, and $s\in Z_{q}^{n}$ be an arbitrary vector that is a secret key of any user. (a,b) is an LWE sample, where $a\in Z_{q}^{n}$ is selected randomly and uniformly, $b\equiv \langle s,a\rangle +e\;\left(\mathrm{mod}\;q\right)$, e←χ.

Let $D_{q}=\left\lfloor q/4\right\rfloor$, $1\leq \tau \leq D_{q}/2$, $a\leftarrow Z_{q}^{n}$, and compute $b\equiv \langle s,a\rangle +e+xD_{q}\;\left(\mathrm{mod}\;q\right)$ for encrypting one bit, $e\in \left[-\tau ,\tau \right]$. Let $E_{s}\left(x\right)=\left(a,b\right)\in Z_{q}^{n}\times Z_{q}$, (a,b) is the LWE ciphertext for x∈{0,1}. Note that $D_{q}=\left\lfloor q/2\right\rfloor$ in Regev’s but $D_{q}=\left\lfloor q/4\right\rfloor$ in SGFHE scheme for homomorphic bit operations.

Modulus reduction can reduce the LWE ciphers of $Z_{q}$ to $Z_{r}$ where *r* is far less than *q*.

**Lemma** **1**  ([7])**.** *Let $s,a\in Z_{q}^{n}$, $e\in Z_{q}^{n}$ with |e|≤τ, $D_{r}=\left\lfloor r/4\right\rfloor$, and $b\equiv \langle s,a\rangle +e+xD_{q}\;\left(\mathrm{mod}\;q\right).$*

*(1) Suppose τ∈q(n−3)/(2r),q≥4r and $s\in {\left\{0,1\right\}}^{n}$. $b^{\prime }=\left\lfloor rb/q\right\rceil ,a^{\prime }=\left\lfloor ra/q\right\rceil$; then*

$$b^{\prime }\equiv \langle s,a^{\prime }\rangle +e+xD_{r}\;\left(\mathrm{mod}\;r\right).$$

*(2) Let $\ell =\left\lceil log_{2}^{q}\right\rceil$, q≥16. Suppose that τ≤q(nℓ−5)/(2r) with $s\leftarrow Z_{q}^{n}$. Then there exist $s^{\prime }\in {\left\{0,1\right\}}^{nl},\;a^{\prime }\in Z_{r}^{n\ell }$ and $b^{\prime }\in Z_{r}$, satisfying*

$$b^{\prime }\equiv s^{\prime }{\left(a^{\prime }\right)}^{t}+e^{\prime }+xD_{r}\;\left(\mathrm{mod}\;r\right),$$

*where $e^{\prime }\in Z$, $|e^{\prime }|\leq n\ell$.*

### 2.3. RLWE Ciphers

Lyubashevsky et al. introduced the RLWE problem to acquire more efficient encryption schemes [33]. An RLWE sample $v=\left(a\left(x\right),b\left(x\right)\right)\in R_{n}^{2}$, where $a\left(x\right)\leftarrow R_{n,q},a\left(x\right)=\sum_{i=0}^{n-1}a_{i}x^{i}$, and

$$b\left(x\right):=s\left(x\right)a\left(x\right)+e\left(x\right)\;(\mathrm{mod}\;\left(x^{n}+1,q\right))$$

for some $e\left(x\right)\leftarrow R_{n}$, ${\left||e\left(x\right)|\right|}_{\infty }\leq \tau$, τ is the bound of error.

$$v{\left(-s\left(x\right),1\right)}^{t}\equiv e\left(x\right)\;\left(\mathrm{mod}\;\left(x^{n}+1,q\right)\right).$$

Let $m\left(x\right)=\sum_{i=0}^{n-1}m_{i}x^{i}$, where $m_{i}\in \left\{0,1\right\}$ denotes an *n*-bit message. The RLWE cipher of m(x) with error size τ is

$$RE_{s}\left(m\left(x\right)\right)=v+m\left(x\right)D_{q}\left(0,1\right)\in R_{n,q}^{2}.$$

Suppose $RE_{s}\left(m\left(x\right)\right)=\left(a\left(x\right),b\left(x\right)\right)$. We have

$$b\left(x\right)-s\left(x\right)a\left(x\right)\equiv m\left(x\right)D_{q}+e\left(x\right)\;\left(\mathrm{mod}\;\left(x^{n}+1,q\right)\right),$$

when $\tau \leq D_{q}/2$, the message m(x) can be recovered from $m\left(x\right)\equiv b\left(x\right)-s\left(x\right)a\left(x\right)\;(\mathrm{mod}\;\left(x^{n}+1,q\right))$.

### 2.4. GSW Ciphers and External Product

#### 2.4.1. Gadget Matrix

Suppose that *B* and *l* are positive integers so that $B^{\ell }\geq q$. Suppose that when $g=(1,B,\cdots ,B^{\ell -1})$, an arbitrary $a\in Z_{q}$ could be denoted by

$$a=a_{0}+a_{1}+\cdots +a_{\ell -1}B^{\ell -1}=\left(a_{0}+a_{1},\cdots +a_{\ell -1}\right)g^{t},$$

where $a_{i}\in Z$ has a small size. Let $-B/2\leq a_{i}\leq B/2$; then $\left(a_{0}+a_{1},\cdots +a_{\ell -1}\right)$ is unique. Let $-2B\leq a_{i}\leq 2B$; the lemma as following is straightforward to prove.

**Lemma** **2** ([7])**.** *Let $B^{\ell }\geq q$, a∈Z. For 0≤i≤ℓ−1, choose $x_{i}\leftarrow Z,\left|x_{i}\right|\leq 3B/2$, which is uniform, random and independent. Suppose that*

$$\begin{array}{c} \;\;\;\;a-(x_{0}+x_{1}B+\cdots +x_{\ell -1}B^{\ell -1})\\ \equiv y_{0}+y_{1}B+\cdots +y_{\ell -1}B^{\ell -1}\;\left(\mathrm{mod}\;q\right) \end{array}$$

*where $|y_{i}|\leq B/2$. Set $a_{i}=x_{i}+y_{i}$; then $\left(a_{0},a_{1},\cdots ,a_{\ell -1}\right)$ is uniform random solution to*

$$a\equiv a_{0}+a_{1}B+\cdots +a_{\ell -1}B^{\ell -1}\;\left(\mathrm{mod}\;q\right)$$

*with $|a_{i}|\leq B/2$.*

Hence, any list of elements in $Z_{q}$ can be extended. That is, each polynomial $a\left(x\right)\in R_{n,q}$ can be denoted by

$$\begin{array}{cc} a(x) & =a_{0}\left(x\right)+a_{1}\left(x\right)B+\cdots +a_{\ell -1}\left(x\right)B^{\ell -1}\\ \; & =\left(a_{0}\left(x\right),a_{1}\left(x\right),\cdots ,a_{\ell -1}\left(x\right)\right)g^{t}, \end{array}$$

where $\left|\right|a_{i}\left(x\right){\left|\right|}_{\infty }\leq 2B$. A gadget matrix of (2ℓ)×2 is defined as

$$G=\left(\begin{array}{cc} g^{t} & 0\\ 0 & g^{t} \end{array}\right).$$

Any $\left(a\left(x\right),b\left(x\right)\right)\in R_{n,q}^{2}$ can be denoted by

(1) (a(x),b(x))=u(x)G

where $u\left(x\right)\in R_{n}^{2\ell }$ is selected randomly and uniformly, and ${\left||u\left(x\right)|\right|}_{\infty }\leq 2B$. Here $G^{-1}$, only as an operator, acts on the right of (a(x),b(x))(*G* is not a square matrix, so it has no inverse).

$$u\left(x\right)=\left(a\left(x\right),b\left(x\right)\right)◃G^{-1}$$

A row vector u(x) has 2ℓ polynomials; the coefficients of the polynomials are small and at most 2B. This can increase the dimension to decease the coefficient. By the above definition, we have the following equation.

$$\left(v◃G^{-1}\right)G=v,\forall v\in R_{n,q}^{2}$$

#### 2.4.2. External Product

Suppose that a row vector $v=\left(a\left(x\right),b\left(x\right)\right)\in R_{n,q}^{2}$, and arbitrary matrices $A\in R_{n,q}^{2\ell \times 2}$ of 2ℓ×2, define the external product of v and *A* as

$$v⊙A=\left(v◃G^{-1}\right)A\in R_{n,q}^{2};$$

it is a random vector; for $v◃G^{-1}$ is a random vector of 1×2ℓ. By definition, the external product satisfies the right distributive, namely, for arbitrary two matrices $A,B\in R_{n,q}^{(2\ell )\times 2}$ of 2ℓ×2, we have

$$\begin{array}{cc} v⊙(A+B) & \equiv \left(v◃G^{-1}\right)\left(A+B\right)\\ \; & =\left(v◃G^{-1}\right)A+\left(v◃G^{-1}\right)B\\ \; & =v⊙A+v⊙B\;(\mathrm{mod}\;\left(x^{n}+1,q\right)). \end{array}$$

#### 2.4.3. GSW Ciphers

Let an *n*-bit secret key $s\left(x\right)=\sum_{i=0}^{n-1}s_{i}x^{i}$, where $s_{i}\in \left\{0,1\right\}$, RLWE sample $A\leftarrow R_{n,q}^{2\ell \times 2}$ (the rows of *A* are RLWE samples) and a GSW cipher for $m\left(x\right)\in R_{n}$ is

$$GSW_{s}\left(m\left(x\right)\right)=A+m\left(x\right)G\in R_{n,q}^{2\ell \times 2};$$

according to the definition of RLWE sample

$$A{\left(-s\left(x\right),-1\right)}^{t}\equiv w\left(x\right)\;\left(\mathrm{mod}\;\left(x^{n}+1,q\right)\right),$$

where $w\left(x\right)\in R_{n}^{2\ell }$, and ||w(x)||≤τ; τ is the error size of GSW ciphers.

**Lemma** **3** ([7])**.** *Let $m_{0}\left(x\right),m_{1}\left(x\right)\in R_{n}$ be any two polynomials. For any $RE_{s}\left(m_{0}\left(x\right)\right)$ with error size τ and any $GSW_{s}\left(m_{1}\left(x\right)\right)$ with error size $\tau_{1}$, we have*

$$RE_{s}\left(m_{0}\left(x\right)\right)⊙GSW_{s}\left(m_{1}\left(x\right)\right)=RE_{s}\left(m_{0}\left(x\right)m_{1}\left(x\right)\right)$$

*and $RE_{s}\left(m_{0}\left(x\right)m_{1}\left(x\right)\right)$ which has an error size of at most $\tau ||m_{1}\left(x\right){\left|\right|}_{\infty }+4Bn\ell \tau_{1}$.*

### 2.5. Bloom Filter

A Bloom filter [34] is a compact data structure for probabilistic set membership testing, and can insert and query data efficiently. The Bloom filter provides a time and space-efficient method to check whether there is an element in the set. A Bloom filter consists of a binary vector and a set of hash functions; $b_{j}$ represents the *j*-th bit of the Bloom filter *b* and all elements of the empty Bloom filter are 0. Any Bloom filter *b* includes the three steps as follows:

Create(α): Create an empty Bloom filter with α bits; the hash function $\left\{h_{i}|0\leq i<\beta \right\}$ is:

$$h_{i}:{\left\{0,1\right\}}^{*}\to \left\{0,\ldots ,\alpha -1\right\}.$$

Add(x): Compute β hash values $g_{i}=h_{i}\left(x\right)$ of the element *x* using the hash function $h_{i}\left(0\leq i<\beta \right)$. Set the Bloom filter cell with subscript $g_{i}$ to 1.

$$g_{i}=h_{i}\left(x\right)⟹b_{g_{i}}=1$$

Test(x): Test whether the element *x* is in the Bloom filter *b*. Compute β hash values $g_{i}=h_{i}\left(x\right)$ of the element *x*; if the β cells with subscript $g_{i}$ are 1 ($b_{g_{i}}=1$), then return 1 (true).

(2) $$Test\left(x\right)=\overset{\beta -1}{\underset{i=0}{\wedge }}b_{h_{i}\left(x\right)}=\overset{\beta -1}{\underset{i=0}{\wedge }}b_{g_{i}}$$

The Bloom filter has a negligible false positive probability; Test(x) will return 1, although *x* cannot be added to the Bloom filter. Given ω elements to be added and the expected maximum false positive probability $2^{-k}$, the Bloom filter size α needs to satisfy:

$$\alpha \geq \frac{\omega k}{ln^{2}2}.$$

A Bloom filter is widely used in cryptography. Bellovin and Cheswick [35] and Goh [36] implemented a securely document search using a Bloom filter. Raykov and Bellovin [37] realized a secure database query. Qiu L and Li Y [38] realized privacy data mining and BIP-0037 put forward the application of a Bloom filter in Bitcoin. Reference [39,40,41] realized the set intersection computing based on Bloom filters.

## 3. Homomorphic Operations

In SGFHE scheme, let any two LWE ciphers be $E_{s}\left(x_{1}\right)$ and $E_{s}\left(x_{2}\right)$ with $x_{1},x_{2}\in \left\{0,1\right\}$; one bootstrapping can compute three bit operations $E_{s}\left(x_{1}\wedge x_{2}\right),E_{s}\left(x_{1}\vee x_{2}\right)$ and $E_{s}\left(x_{1}⊕x_{2}\right)$; the scheme follows the approach in Ducas et al. [42] and Chillotti [43], but does not need to perform a key switch.

### 3.1. Key Generations

Let *n* be a power of 2, n≥64. Suppose that *r* can be divided by 8; $m=r/2,B=35r^{2}n$,

(3) $$r\geq 16n,q\geq nr,1220r^{4}n^{2}\leq Q<1225r^{4}n^{2}=B^{2}.$$

$$D_{r}=\left\lfloor r/4\right\rfloor ,D_{q}=\left\lfloor q/4\right\rfloor ,{\tilde{D}}_{Q}=\left\lfloor Q/8\right\rfloor ,and\;G=\left(\begin{array}{cc} 1 & 0\\ B & 0\\ 0 & 1\\ 0 & B \end{array}\right).$$

**Secret key** Pick $s\leftarrow {\left\{0,1\right\}}^{n}$ uniformly and randomly; let $s\left(x\right)=\sum_{i=0}^{n-1}s_{i}x^{i}$.

**Public key**$pk=\left(k_{0}\left(x\right),k_{1}\left(x\right)\right),k_{0}\left(x\right)\leftarrow R_{n,q}$,

$$k_{1}\left(x\right)\equiv k_{0}\left(x\right)s\left(x\right)+e\left(x\right)\;\left(\mathrm{mod}\;x^{n}+1,q\right),$$

where $e\left(x\right)\leftarrow R_{n}{,||e\left(x\right)||}_{\infty }<D_{q}/\left(41n\right)$.

**Bootstrapping key** A bootstrapping key $bk=(C_{0},C_{1},\ldots ,C_{n-1})$ can be generated as follows:

For 1≤i≤n−1 do:

(1) Pick $a_{ji}\leftarrow R_{m,Q}$, 1≤j≤4;
(2) Pick $e_{ji}\left(x\right)\in R_{m}$, $\left|\right|e_{ji}{\left|\right|}_{\infty }\leq n$, 1≤j≤4;
(3) Compute $b_{ji}\left(x\right):=a_{ji}\left(x\right)s\left(x\right)+e_{ji}\left(x\right)\;\left(\mathrm{mod}\;x^{m}+1,Q\right)$, 1≤j≤4;
(4) Set $C_{i}:=\left(\begin{array}{cc} a_{1i}\left(x\right) & b_{1i}\left(x\right)\\ a_{2i}\left(x\right) & b_{3i}\left(x\right)\\ a_{3i}\left(x\right) & b_{2i}\left(x\right)\\ a_{4i}\left(x\right) & b_{4i}\left(x\right) \end{array}\right)+s_{i}G\;\left(\mathrm{mod}\;Q\right)$.

### 3.2. Bootstrapping Algorithm

**Lemma** **4.** *Suppose that a bootstrapping key bk has an error size at most $\tau_{1}$; r is divisible by 8 and $r\geq \;16n,Q\;\geq \;\frac{n}{n-3}16Br^{2}\ell \tau_{1}$. Then, for any two LWE ciphers $E_{s}\left(x_{i}\right)=v_{i}\in Z_{r}^{n}\times Z$, with error size $\leq D_{r}/4$ where $x_{i}\in \left\{0,1\right\}$ for i=1,2, the bootstrapping algorithm in Algorithm 1 outputs random LWE ciphers $E_{s}\left(x_{1}\wedge x_{2}\right),E_{s}\left(x_{1}\vee x_{2}\right),E_{s}\left(x_{1}⊕x_{2}\right)\in Z_{r}^{n}\times Z_{r}$ all with error size $<n\leq D_{r}/4$ [7].*

**Algorithm 1** Bootstrapping Algorithm: $BT_{bk}\left(v_{1},v_{2}\right)\to c_{1},c_{2},c_{3}$.**Input:**$bk=\left(C_{0},C_{1},\ldots ,C_{n-1}\right)\in R_{m,Q}^{2\ell \times 2}$: bootstrapping key;   $\left(v_{1},v_{2}\right)\in Z_{r}^{n}\times Z_{r}$:$v_{i}=E_{s}\left(x_{i}\right),x_{1},x_{2}\in \left\{0,1\right\}$;**Output:**$E_{s}\left(x_{1}\wedge x_{2}\right),E_{s}\left(x_{1}\vee x_{2}\right),E_{s}\left(x_{1}⊕x_{2}\right)\in Z_{r}^{n}\times Z_{r}$;1:Compute $u:=v_{1}+v_{2}=\left(u_{0},u_{1},\ldots ,u_{n-1},u_{n}\right)\in Z_{r}^{n}\times Z_{r}$;2:$T:=\left\{j\in Z:-D_{r}\leq j\leq D_{r}\right\},t\left(x\right):=\sum_{j\in T}x^{j}$;3:$A:=\left(0,t\left(x\right)x^{-u_{n}}{\tilde{D}}_{Q}\right)\in R_{m,Q}^{2}$;4:**for**kfrom0ton−1**do**5:  $A:=A⊙(G+\left(x^{u_{k}}-1\right)C_{k})$;6:**end for**7:Let $A=(\sum_{i=0}^{m-1}a_{i}x^{i},\sum_{i=0}^{m-1}b_{i}x^{i})$. Set $a_{1}:=\left(Extract\left(a\left(x\right),3m/4\right),{\tilde{D}}_{Q}+b_{3m/4}\right)\in Z_{Q}^{n}\times Z_{Q}$; $a_{2}:=\left(Extract\left(a\left(x\right),m/4\right),{\tilde{D}}_{Q}-b_{m/4}\right)\in Z_{Q}^{n}\times Z_{Q}$; $a_{3}:=a_{2}-a_{1}\in Z_{Q}^{n}\times Z_{Q}$;8:**for**ifrom1to3**do**9:  $c_{i}:=\left\lfloor ra/Q\right\rceil \in Z_{r}^{n}\times Z_{r}$;10:**end for**11:Return $c_{1},c_{2},c_{3}$;

### 3.3. Encryption Scheme

**Lemma** **5.** *Suppose that $r=2^{t+1}$; $\left(a\left(x\right),b\left(x\right)\right)\in R_{n,r}^{2}$ can be computed from Algorithm 2. Then for some $\omega_{3}\left(x\right),||\omega_{3}\left(x\right){\left|\right|}_{\infty }\leq D_{r}/4$, so that*

$$2^{t-4}b\left(x\right)-s\left(x\right)a\left(x\right)\equiv \omega_{3}\left(x\right)+m\left(x\right)D_{r}\;\left(\mathrm{mod}\;x^{n}+1,r\right).$$

*Specifically, if r=16n, then (u,v) returned in Algorithm 2 has 6n bits and represents an RLWE cipher $RE_{s}\left(m\left(x\right)\right)$, and the error size <n [7].*

**Algorithm 2** Encryption with private key:$RE_{s}\left(m\left(x\right)\right)\to (u,v$).**Input:***n*-bit secret key $s\left(x\right)=\sum_{i=0}^{n-1}s_{i}x^{i},\;s_{i}\in \left\{0,1\right\}$;   *n*-bit message
$m\left(x\right)=\sum_{i=0}^{n-1}m_{i}x^{i},\;m_{i}\in \left\{0,1\right\}$;   $t:=\left\lceil log_{2}\left(r\right)\right\rceil$, hence $2^{t}\leq r\leq 2^{t-1}$;   $P:\left\{0,1\right\}\to {\left\{0,1\right\}}^{n(t+1)}$;
**Output:**
$\left(u,v\right)\in {\left\{0,1\right\}}^{n}\times {\left\{{\left\{0,1\right\}}^{5}\right\}}^{n}$

1:$u\leftarrow {\left\{0,1\right\}}^{n},\;a\left(x\right):=P\left(u,x\right)\in R_{n,r}$;2:$\omega \left(x\right)\leftarrow R_{n}{,||\omega \left(x\right)||}_{\infty }\leq D_{r}/8,\;b_{1}\left(x\right):=a\left(x\right)s\left(x\right)+\omega \left(x\right)+m\left(x\right)D_{r}\;\left(\mathrm{mod}\;x^{n}+1,r\right)$;3:$b\left(x\right)=\sum_{i=0}^{n-1}b_{i}x^{i}:=\left\lfloor b_{1}\left(x\right)/2^{t-4}\right\rfloor$;4:$v=\left(b_{1},b_{2},\ldots ,b_{n-1}\right)\in {\left({\left\{0,1\right\}}^{5}\right)}^{n}$;5:return (u,v);

**Lemma** **6.** *Suppose that $r=2^{t+1},\;r\geq 16n,\;q\geq 4r\;and\;n\geq 164$. Suppose that $RE_{pk}\left(m\left(x\right)\right):=\left(a\left(x\right),b\left(x\right)\right)\in R_{n,r}^{2}$ be any ciphertext output by Algorithm 3. Then $2^{t-5}b\left(x\right)-s\left(x\right)a\left(x\right)\equiv \omega_{3}\left(x\right)+m\left(x\right)D_{r}\;\left(\mathrm{mod}\;x^{n}+1,r\right)$ for some $\omega_{3}\left(x\right)\in R_{n}$ with $\left|\right|\omega_{3}\left(x\right){\left|\right|}_{\infty }\leq D_{r}/4$.*

*Specifically, if r=16n, then any ciphertext (a(x),b(x)) has $n(10+log_{2}\left(n\right))$ bits and the error, that is, each coefficient of $\omega_{3}\left(x\right)$, is in (−n,n) randomly [7].*

We can divide the data x into *d* blocks of length *n*. Let N=dn, $x=\left(x_{1},x_{2},\ldots ,x_{d}\right)\in {\left\{0,1\right\}}^{N}$, $x_{k}=\left(x_{k,0},x_{k,1},\cdots ,x_{k,n-1}\right),x_{k}\in {\left\{0,1\right\}}^{n}$. Each $x_{k}$ can be expressed as a polynomial $\sum_{i=0}^{n-1}x_{k,i}x^{i}\in R_{n}$. Then—encrypted using the private-key scheme $c_{k}=RE_{s}\left(x_{k}\right),1\leq k\leq d$ by Algorithm 2—note that the cipher text size $c_{k}$ is about 6N bits and then encrypted using the public-key scheme $c_{k}^{\prime }=RE_{pk}\left(x_{k}\right),1\leq k\leq d$ by Algorithm 3; note that the cipher text size $c_{k}$’ is about $N(10+log_{2}\left(n\right))$. Homomorphic computing can be performed in three steps as follows:

**Algorithm 3** Encryption under public key: $RE_{pk}\left(m\left(x\right)\right)\to \left(a\left(x\right),b\left(x\right)\right)\in R_{n,r}^{2}$.**Input:**$pk=\left(k_{0}\left(x\right),k_{1}\left(x\right)\right),k_{0}\left(x\right)\leftarrow R_{n,q}$;   $m\left(x\right)=\sum_{i=0}^{n-1}m_{i}x^{i}$:*n*-bit message where each   $m_{i}\in \left\{0,1\right\}$;
   $t:=\left\lceil log_{2}\left(r\right)\right\rceil$;**Output:**$\left(a\left(x\right),b\left(x\right)\right)\in R_{n,r}^{2}$$u\left(x\right)\leftarrow R_{n}$,
1:$u\left(x\right)\leftarrow R_{n}$, each coefficient random from {−1,0,1};2:$\omega_{1}\left(x\right)\leftarrow R_{n},||\omega_{1}\left(x\right){\left|\right|}_{\infty }\leq D_{q}/\left(41n\right)$;3:$\omega_{2}\left(x\right)\leftarrow R_{n},||\omega_{2}\left(x\right){\left|\right|}_{\infty }\leq D_{q}/82$;4:$a_{1}\left(x\right):=k_{0}\left(x\right)u\left(x\right)+\omega_{1}\left(x\right)\;\left(\mathrm{mod}\;x^{n}+1,q\right)$;5:$b_{1}\left(x\right):=k_{1}\left(x\right)u\left(x\right)+\omega_{2}\left(x\right)+m\left(x\right)D_{q}\;\left(\mathrm{mod}\;x^{n}+1,q\right)$;6:$a\left(x\right):=\left\lfloor \frac{r}{q}a_{1}\left(x\right)\right\rceil ,b\left(x\right):=\left\lfloor \frac{r}{2^{t-5}q}b_{1}\left(x\right)\right\rceil$;7:Return (a(x),b(x))

(1) Unpacking the RLWE ciphertexts $RE(x_{k})$ to get LWE ciphers in $Z_{r}^{n}\times \;Z_{r}$ for the bits of x.
  $$RE\left(x_{k}\right)\overset{unpack}{⟶}E_{s}\left(c_{k,i}\right)$$
(2) Homomorphic computing of $f\left(x\right)=y=\left\{y_{0},y_{1},\ldots ,y_{M}\right\}\in {\left\{0,1\right\}}^{M}$ on LWE ciphers.
  $$f\left(x\right)\overset{BT_{bk}}{⟶}\left\{E_{s}\left(y_{0}\right),E_{s}\left(y_{1}\right),\ldots ,E_{s}\left(y_{M}\right)\right\}$$
(3) Packing the LWE ciphers $\left\{E_{s}\left(y_{0}\right),E_{s}\left(y_{1}\right),\ldots ,E_{s}\left(y_{M}\right)\right\}$ of function *f* into RLWE ciphers in $R_{n,r}^{2}$.
  $$\left\{E_{s}\left(y_{0}\right),E_{s}\left(y_{1}\right),\ldots ,E_{s}\left(y_{M}\right)\right\}\overset{pack}{⟶}RE_{s}\left(y\right)$$

## 4. Privacy-Preserving Set Intersection

We abstract the privacy set intersection computation model as follows. The client *C* owns a set $\left\{c_{1},\ldots ,c_{v}\right\}$ of size *v*, and the server *S* holds a set $\left\{s_{1},\ldots ,s_{\omega }\right\}$ of size ω. After the end of the protocol, the client *C* only obtains the intersection $\left\{c_{1},\ldots ,c_{v}\right\}⋂\left\{s_{1},\ldots ,s_{\omega }\right\}$; however, the server cannot get any information for the input and the set intersection of the client (including the size of the intersection).

### 4.1. The Basic Two-Party Computing Protocol

**Figure 1:**
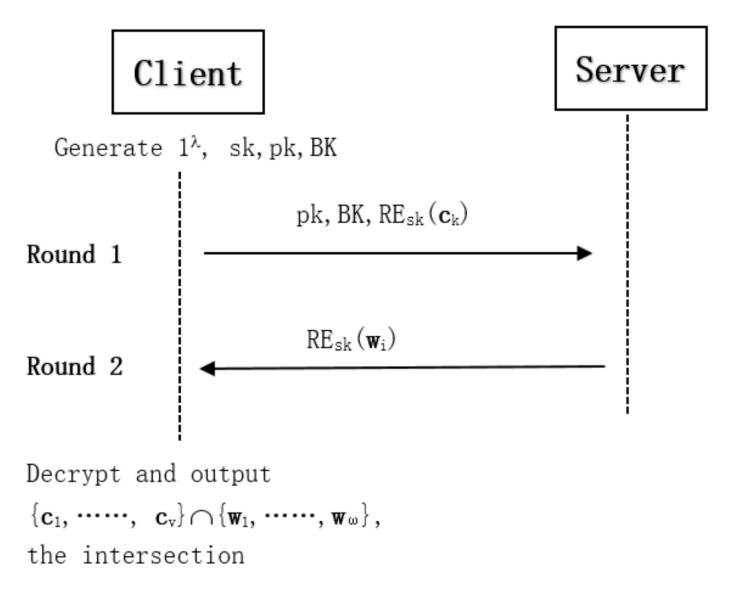
Summary of the intersection protocol.

The summary of basic private two-party intersection protocol is shown in Figure 1. The specific steps are as follows:

1. The client *C* encrypts the set with private key and sends ciphertexts to the server *S*.
2. The server *S* implements homomorphic computing with bootstrapping key and sends the result to the client *C*.
3. The client *C* decrypts and computes the intersection of the two sets; the server *S* cannot acquire any information about the input and output.

**Figure 2:**
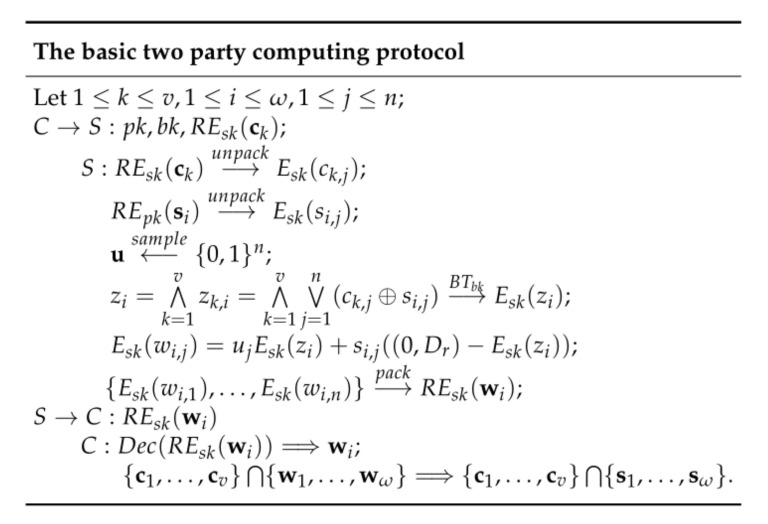
The basic two-party computing protocol.

Our basic two-party computing protocol is shown in Figure 2. At step C→S, the client sends pk,bk and $RE_{sk}\left(c_{k}\right)$ to the server. At step S, the server unpacks $RE_{sk}\left(c_{k}\right)$ to get $E_{sk}\left(c_{k,j}\right)$, unpacks $RE_{pk}$ to get $E_{sk}\left(s_{i,j}\right)$, samples $u\in {\left\{0,1\right\}}^{n}$, calls bootstrapping operations to compute $E_{sk}\left(z_{k,i}\right)$, computes LWE ciphers $E_{sk}\left(w_{i,j}\right)$, packs the resulted LWE ciphers $E_{sk}\left(w_{i,j}\right)$ into RLWE ciphers $RE_{sk}\left(w_{i}\right)$ and sends them to the client. At step C, the client decrypts $RE_{sk}\left(w_{i}\right)$ to get $w_{i}$ and computes the intersection.

### 4.2. Correctness of the Basic Two-Party Computing Protocol

First, the correctness of SGFHE scheme has been proven.

Let $c_{k},s_{i}$ be the set elements’ binary representation of the client and server respectively. The insufficient bits are filled with 0s and we extend the length to *n*.

$$c_{k}=\left\{c_{k,1},\ldots ,c_{k,n}\right\}={\left\{0,1\right\}}^{n},1\leq k\leq v$$

$$s_{i}=\left\{s_{i,1},\ldots ,s_{i,n}\right\}={\left\{0,1\right\}}^{n},1\leq i\leq \omega$$

$$u\overset{sample}{⟵}{\left\{0,1\right\}}^{n}$$

(4) $$z_{k,i}=\overset{n}{\underset{j=1}{⋁}}\left(c_{k,j}⊕s_{i,j}\right)$$

If $z_{k,i}=1$, then $c_{k}\neq s_{i}$; if $z_{k,i}=0$, then $c_{k}=s_{i}$.

The server can acquire $E_{sk}\left(z_{k,i}\right)$ by $RE_{sk}\left(c_{k}\right)\overset{unpack}{⟶}E_{sk}\left(c_{k,j}\right),RE_{pk}\left(s_{i}\right)\overset{unpack}{⟶}E_{sk}\left(s_{i,j}\right)$ and call (2n−1) bootstrapping operations, denoted by $z_{k,i}=\overset{n}{\underset{j=1}{⋁}}\left(c_{k,j}⊕s_{i,j}\right)\overset{BT_{bk}}{⟶}E_{sk}\left(z_{k,i}\right)$.

*Remark: RE represents RLWE cipher; E represents LWE cipher.*

Let

(5) $$z_{i}=\overset{v}{\underset{k=1}{⋀}}z_{k,i};$$

$E_{sk}\left(z_{i}\right)\in Z_{r}^{n}\times Z_{r}$ can be computed from $E_{sk}\left(z_{k,i}\right)$ by implementing (v−1) bootstrapping operations. Hence, implementing (2n+v−2) bootstrapping operations by (6) can compute $E_{sk}\left(z_{i}\right)$.

(6) $$z_{i}=\overset{v}{\underset{k=1}{⋀}}z_{k,i}=\overset{v}{\underset{k=1}{⋀}}\overset{n}{\underset{j=1}{⋁}}\left(c_{k,j}⊕s_{i,j}\right)\overset{BT_{bk}}{⟶}E_{sk}\left(z_{i}\right)$$

If $z_{i}=1$, then $w_{i}=u$ is a random value with $\forall k,c_{k}\neq s_{i}$; if $z_{i}=0$, then there ∃k so that $c_{k}=s_{i},w_{i}=c_{k}=s_{i}$ is in the intersection. For $s_{i}$ and u, each bit

$$w_{i,j}=z_{k}\wedge u_{j}⊕\left(1-z_{k}\right)\wedge s_{i,j}$$

can be computed by

(7) $$w_{i}=\left\{w_{i,1},\ldots ,w_{k,n}\right\}=z_{i}u⊕\left(1-z_{i}\right)s_{i}$$

For plaintexts $u_{j}$ and $s_{i,j}$, an LWE cipher of any bit $z_{k}\wedge u_{j}⊕\left(1-z_{k}\right)\wedge s_{i,j}$ can be computed as

$$u_{j}E_{sk}\left(z_{i}\right)+s_{i,j}\left(E_{sk}\left(1\right)-E_{sk}\left(z_{k}\right)\right),$$

which still has error size $<D_{r}/4$.

The LWE cipher is

(8) $$E_{sk}\left(w_{i,j}\right)=u_{j}E_{sk}\left(z_{i}\right)+s_{i,j}\left(E_{sk}\left(1\right)-E_{sk}\left(z_{k}\right)\right).$$

The server can pack the resulted LWE ciphers $E_{sk}\left(w_{i,j}\right)$ into RLWE ciphers $RE_{sk}\left(w_{i}\right)$ and send them to the client.

In the end, the client decrypts $Dec\left(RE_{sk}\left(w_{i}\right)\right)⟹w_{i}$ and computes the intersection

$\left\{c_{1},\ldots ,c_{v}\right\}⋂\left\{w_{1},\ldots ,w_{\omega }\right\}⟹$ $\left\{c_{1},\ldots ,c_{v}\right\}⋂\left\{s_{1},\ldots ,s_{\omega }\right\}.$

### 4.3. Security Analysis of the Basic Two-Party Computing Protocol

We analyze the security of the protocol by comparing the real model and the ideal model. The real model is the actual implementation of the basic private intersection protocol and it is a trusted server for computing the intersection. The trusted server receives the input $\left\{c_{1},\ldots ,c_{v}\right\}$ of the client and the input $\left\{s_{1},\ldots ,s_{\omega }\right\}$ of the server, and will return the intersection with the client; however, the server cannot get any information about the output. The ideal model maintains all security evidence. In the semi-honest model, the participant’s view includes its own input and the information received from other participants during the progression of the protocol. The simulator can use the participant’s input and output to build a simulation that is computationally indistinguishable from the views. That proves that the participants cannot obtain any other information besides the inputs and outputs.

**Theorem** **1.** *If SGFHE is held, then the basic two-party computing protocol can realize the private set intersection computing under the semi-honest model.*

**Proof.**  In the protocol, the server cannot obtain any other information besides receiving the RLWE ciphers. Its view can only be simulated with ciphertexts and its security is based on IND-CPA security of RLWE scheme.

The client only receives the RLWE ciphers of the intersections and the random RLWE ciphers. Therefore, it just includes the output information of the set intersection and the view of simulator is only the output information of the set intersection. □

### 4.4. The Improvement of the Basic Two-Party Computing Protocol

**Figure 3:**
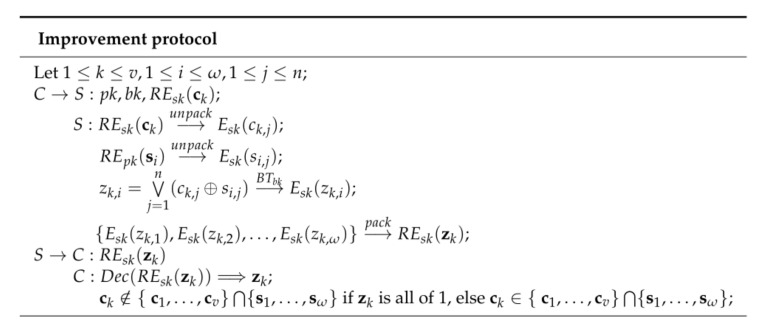
Improvement.

In the basic two-party computing protocol, the server will return the ciphertexts of the intersection elements or the random ciphertexts, and computes the intersection by decrypting the ciphertexts. In our improvement protocol shown in Figure 3, we just need to determine whether $c_{k}$ is in $\left\{s_{1},\ldots ,s_{\omega }\right\}$ without computing the ciphertexts of the intersection elements by the server. On the one hand, it can reduce the computational complexity; on the other hand, it will not reveal the size of the server set.

Let $c_{k},s_{i}$ be the set elements’ binary representations of the client and the server respectively. The insufficient bits are filled with 0s and we extend the length to *n*.

$$c_{k}=\left\{c_{k,1},\ldots ,c_{k,n}\right\}={\left\{0,1\right\}}^{n},1\leq k\leq v$$

$$s_{i}=\left\{s_{i,1},\ldots ,s_{i,n}\right\}={\left\{0,1\right\}}^{n},1\leq i\leq \omega$$

(9) $$z_{k,i}=\overset{n}{\underset{j=1}{⋁}}\left(c_{k,j}⊕s_{i,j}\right)$$

If $z_{k,i}=1$, then $c_{k}\neq s_{i}$; if $z_{k,i}=0$, then $c_{k}=s_{i}$.

The server can acquire $E_{sk}\left(z_{k,i}\right)$ by $RE_{sk}\left(c_{k}\right)\overset{unpack}{⟶}E_{sk}\left(c_{k,j}\right)$, $RE_{pk}\left(s_{i}\right)\overset{unpack}{⟶}E_{sk}\left(s_{i,j}\right)$ and call (2n−1) bootstrapping operations, denoted by $z_{k,i}=\overset{n}{\underset{j=1}{⋁}}\left(c_{k,j}⊕s_{i,j}\right)\overset{BT_{bk}}{⟶}E_{sk}\left(z_{k,i}\right)$. The server packs LWE ciphers $E_{sk}\left(z_{k,i}\right)$ to RLWE ciphers $RE_{sk}\left(z_{k}\right)$ and sends them to the client.

$$\left\{E_{sk}\left(z_{k,1}\right),E_{sk}\left(z_{k,2}\right),\ldots ,E_{sk}\left(z_{k,\omega }\right)\right\}\overset{pack}{⟶}RE_{sk}\left(z_{k}\right),$$

the client decrypts $RE_{sk}\left(z_{k}\right)$, if all $z_{k}$ is 1, then

$$c_{k}\notin \left\{c_{1},\ldots ,c_{v}\right\}⋂\left\{s_{1},\ldots ,s_{\omega }\right\};$$

else $c_{k}\in \left\{c_{1},\ldots ,c_{v}\right\}⋂\left\{s_{1},\ldots ,s_{\omega }\right\}.$

In the protocol, the server cannot obtain any other information besides RLWE ciphers and the view can only be simulated by the ciphertexts. Its security is based on IND-CPA security of RLWE scheme.

The client acquires $z_{k,i}$ by (9), however, the probability of obtaining $s_{i,j}$ from $z_{k,i}$ and $c_{k,j}$ is $2^{-n}$, and it is negligible. The client only receives the output of the intersection; therefore, the view of simulator is just the output of the set intersection.

### 4.5. Two-Party Computing Protocol Based on a Bloom Filter

**Figure 4:**
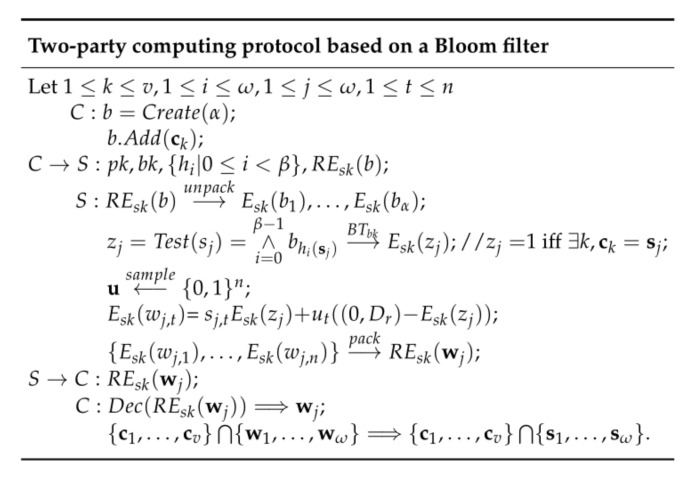
Protocol based on a Bloom filter.

In this section, we construct a two-party protocol based on Bloom filter shown in Figure 4, in which the client *C* encrypts each bit of the Bloom filter with private key and sends it to the server *S*. The server *S* homomorphic computes $Test(s_{j})$ with the bootstrapping key of client *C* and sends it to the client. *C* will obtain the intersection of the two sets by decrypting, but the server cannot get any information about the input and output (including the size of the intersection).

Let $c_{k},s_{i}$ be the set elements’ binary representations of the client and the server respectively. The insufficient bits are filled with 0s and we extend the length to *n*.

$$c_{k}=\left\{c_{k,1},\ldots ,c_{k,n}\right\}={\left\{0,1\right\}}^{n},1\leq k\leq v.$$

$$s_{j}=\left\{s_{j,1},\ldots ,s_{j,n}\right\}={\left\{0,1\right\}}^{n},1\leq j\leq \omega .$$

The client *C* constructs a Bloom filter b=create(α) and sends $pk,bk,RE_{sk}\left(b\right)$ to the server S.

(10) $$z_{j}=Test\left(s_{j}\right)=\overset{\beta -1}{\underset{i=0}{\wedge }}b_{h_{i}\left(s_{j}\right)}$$

According to (10), input $E_{sk}\left(b_{1}\right),\ldots ,E_{sk}\left(b_{\alpha }\right)$,

$$E_{sk}\left(z_{j}\right)=\overset{\beta -1}{\underset{i=0}{\wedge }}E_{sk}\left(b_{h_{i}\left(s_{j}\right)}\right).$$

Call (β−1) bootstrapping operations to obtain $E_{sk}\left(z_{j}\right)$, denoted by

$$z_{j}=Test\left(s_{j}\right)=\overset{\beta -1}{\underset{i=0}{\wedge }}b_{h_{i}\left(s_{j}\right)}\overset{BT_{bk}}{⟶}E_{sk}\left(z_{j}\right).$$

(11) $$w_{j}=\left\{w_{j,1},\ldots ,w_{j,n}\right\}=z_{j}s_{j}⊕\left(1-z_{j}\right)u$$

If $z_{j}=1$, then there ∃k such that $c_{k}=s_{j}$, and computing $w_{j}=s_{j}$ by (11); similarly, if $z_{j}=0$, then ∀k such that $c_{k}\neq s_{j}$, and computing $w_{j}=u$ by (11). For plaintexts $s_{j}$ and u, each bit can be computed by (11),

$$w_{j,t}=z_{j}\wedge s_{j,t}⊕\left(1-z_{j}\right)\wedge u_{t},1\leq t\leq n.$$

The corresponding LWE cipher is

(12) $$E_{sk}\left(w_{j,t}\right)=s_{j,t}E_{sk}\left(z_{j}\right)+u_{t}\left(E_{sk}\left(1\right)-E_{sk}\left(z_{j}\right)\right).$$

The correctness and security of the two-party computing protocol based on Bloom filter is similar to the basic two-party computing protocol. Please refer to Section 4.2 and Section 4.3.

## 5. Conclusions

We constructed the set intersection two-party computing protocols based on a fully homomorphic encryption scheme. The protocols are simple and only need two rounds of communication, and the security is based on RLWE and LWE problems in the semi-honest model. The ciphertext extension of the protocols is small so that the protocols have strong practicability. Furthermore, we can extended the set intersection protocol by outsourcing computing under the malicious model. The limitation of our schemes is they are two-party protocols. In future work, we shall extend them to multi-party protocols. The disadvantage of the private set intersection protocols is they are not efficient enough due to bottleneck the bootstrapping operation. On the theoretical side, with the development of fully homomorphic encryption technology, its performance has been greatly improved, but the efficiency of it is still worthy of in-depth study. The bottleneck of the SGFHE scheme is its bootstrapping operation; therefore, its parallelization and hardware implementation will be further studied to improve the overall efficiency of the protocol.
